# Molecular characterization of extended-spectrum β-lactamase-producing *Escherichia coli* isolated from lower respiratory tract samples between 2002 and 2019 in the Central Slovenia region

**DOI:** 10.1186/s12941-023-00664-1

**Published:** 2024-01-13

**Authors:** Katja Hrovat, Katja Molan, Katja Seme, Jerneja Ambrožič Avguštin

**Affiliations:** 1 Department of Biology, Biotechnical Faculty, University of Ljubljana, Ljubljana, Slovenia; 2 Department of Microbiology, Biotechnical Faculty, University of Ljubljana, Ljubljana, Slovenia; 3 Institute of Microbiology and Immunology, Faculty of Medicine, University of Ljubljana, Ljubljana, Slovenia

**Keywords:** *Escherichia coli*, Lower respiratory tract, Extended-spectrum β-lactamase, Antibiotic resistance, ST131, Virulence factors

## Abstract

**Background:**

Antibiotic resistance is one of the most serious global health problems and threatens the effective treatment of bacterial infections. Of greatest concern are infections caused by extended-spectrum β-lactamase-producing *Escherichia coli* (ESBL-EC). The aim of our study was to evaluate the prevalence and molecular characteristics of ESBL-EC isolated over an 18-year pre-COVID period from lower respiratory tract (LRT) samples collected from selected Slovenian hospitals.

**Objectives and methods:**

All isolates were identified by MALDI-TOF and phenotypically confirmed as ESBLs by a disk diffusion assay. Using a PCR approach, 487 non-repetitive isolates were assigned to phylogroups, sequence type groups, and clonal groups. Isolates were also screened for virulence-associated genes (VAGs) and antimicrobial resistance genes.

**Results:**

The prevalence of ESBL-EC isolates from LRT in a large university hospital was low (1.4%) in 2005 and increased to 10.8% by 2019. The resistance profile of 487 non-repetitive isolates included in the study showed a high frequency of group 1 *bla*_CTX-M_ (77.4%; n = 377), *bla*_TEM_ (54.4%; n = 265) and *aac(6')-Ib-cr* (52%; n = 253) genes and a low proportion of *bla*_SHV_ and *qnr* genes. Isolates were predominantly assigned to phylogroup B2 (73.1%; n = 356), which was significantly associated with clonal group ST131. The ST131 group accounted for 67.6% (n = 329) of all isolates and had a higher number of virulence factor genes than the non-ST131 group. The virulence gene profile of ST131 was consistent with that of other extraintestinal pathogenic *E. coli* (ExPEC) strains and was significantly associated with ten of sixteen virulence factor genes tested. Using ERIC-PCR fingerprinting, isolates with the same ERIC-profile in samples from different patients, and at different locations and sampling dates were confirmed, indicating the presence of “hospital-adapted” strains.

**Conclusion:**

Our results suggest that the ESBL-EC isolates from LRT do not represent a specific pathotype, but rather resemble other ExPEC isolates, and may be adapted to the hospital environment. To our knowledge, this is the first study of ESBL-EC isolated from LRT samples collected over a long period of time.

**Supplementary Information:**

The online version contains supplementary material available at 10.1186/s12941-023-00664-1.

## Introduction

*Escherichia coli* (*E. coli)* is primarily a commensal in the gastrointestinal tract of humans and warm-blooded animals. However, due to its genomic plasticity, strains can exhibit considerable genetic diversity. Endowed with a broad spectrum of different virulence-associated genes (VAGs), they can be involved in a variety of intestinal and extraintestinal diseases. ExPEC strains are the leading cause of urinary tract infections (UTIs), a common cause of bacteremia, and to a lesser extent, the causative agent of neonatal meningitis, respiratory tract infections, skin infections, and soft tissue infections [1–3].

Although respiratory infections caused by *E. coli* are rare in humans, they have increased in recent years. They can be caused by inhalation and/or by aspiration of oropharyngeal, gastric contents and/or upper respiratory tract secretions [4, 5]. In addition, *E. coli* pneumonia often develops in patients with underlying diseases such as chronic obstructive pulmonary disease, and bacteremia due to *E. coli* originating from the urinary or gastrointestinal tract. Respiratory infections are common in long-term care facilities and hospitals, especially in intensive care units [6, 7].

In the last decade, *E. coli* infections have become a serious global health problem due to the emergence and rapid pandemic spread of highly virulent and antimicrobial-resistant clones. The global increase in multidrug-resistant ExPEC strains is often associated with sequence type 131 (ST131), which can account for up to 30% of all clinical *E. coli* isolates and up to 80% of ESBL-EC. Several studies have focused on the genetic characteristics of ESBL-EC isolated from UTIs or bacteremia, and less frequently from the respiratory tract [8–11]. Therefore, the aim of our study was to determine whether ESBL-EC from LRT represent a distinct group of ExPEC strains. We analyzed ESBL-groups, plasmid-mediated quinolone resistance (PMQR) genes, phylogenetic background, prevalence of ST131, clonal diversity, and virulence gene profile among ESBL-EC isolates from LRT collected over an 18-year period from patients treated in hospitals in the Central Slovenia region.

## Materials and methods

### Bacterial strains and patients

According to the available data (Additional file 1.1), between January 2005 and December 2019, more than 4800 *E. coli* isolates were obtained from LRT samples (sputa, tracheal aspirates, and bronchoalveolar lavages) from patients hospitalized in the large university hospital. Isolates were also obtained from a national center of oncology, one general hospital, and some specialized outpatient and community healthcare centers in the Central Slovenia region. The number of isolated *E. coli* from other location is not available. All isolates were isolated and identified at the Institute of Microbiology and Immunology, Faculty of Medicine, University of Ljubljana (IMI), by using matrix-assisted laser desorption/ionization time-of-flight mass-spectrometry (MALDI TOF MS) (MBT COMPASS 4.1, Microflex, Bruker Daltonics, Bremen, Germany).

ESBL-EC isolates included in this study were isolated from clinical LRT specimens obtained from 192 (39.4%) female and 281 (57.7%) male patients with suspected LRT infection. 117 (24%), 345 (70.8%) and 11 (2.3%) strains were isolated from sputa, tracheal aspirates and bronchoalveolar lavages, respectively (Additional file 4). The majority of patients belonged to age groups 71–80 years (n = 114; 23.4%) and 81–90 years (n = 113; 23.2%), followed by age groups 61–70 years (n = 81; 16.6%), 51–60 years (n = 51; 10.5%), 0–10 years (n = 33; 6.8%), 91 years and over (n = 30; 6.2%), 41–50 years (n = 28; 5.7%), 21–30 years (n = 11; 2.3%), 31–40 years (n = 7; 1.4%), 11–20 years (n = 5; 1%). 389 (79.9%) patients were older than 51 years. Due to a change in laboratory information system data on sample origin as well as patient details were not available for 14 (2.9%) strains isolated from 2002 to 2004.

### Antimicrobial susceptibility testing

Phenotypic resistance to antimicrobial agents was determined using the disk diffusion assay (Additional file 4). Antibacterial agents routinely tested over the whole study period included ampicillin, amoxicillin-clavulanic acid, piperacillin-tazobactam, cefuroxime, cefotaxime, ceftriaxone, ceftazidime, cefepime, imipenem, ertapenem, gentamicin, amikacin, ciprofloxacin, levofloxacin, and trimethoprim-sulphametoxazole. Results were interpreted according to Clinical and Laboratory Standards Institute (CLSI) [12] guidelines through March 31, 2014, and European Committee on Antimicrobial Susceptibility Testing (EUCAST) [13] guidelines since April 1, 2014. Extended-spectrum β-lactamase production was tested according to CLSI [12] and EUCAST [14] recommendations in the aforementioned time frame. Based on the phenotypic resistance profiles obtained by disk diffusion assay, we categorized the selected 487 isolates as multidrug resistant (MDR) or extensively drug resistant (XDR). MDR isolates were defined as non-susceptible to at least one agent in three or more antimicrobial categories, and XDR isolates as non-susceptible to at least one agent in all but in two or fewer antimicrobial categories. According to the tested antimicrobials, we considered the following groups as individual antimicrobial categories: penicillins, penicillins + β-lactamase inhibitors, non-extended spectrum cephalosporins, extended spectrum cephalosporins, anti-MRSA cephalosporins, aminoglycosides, monobactams, folate pathway inhibitors, fluoroquinolones, tetracyclines, carbapenems [15, 16] (Additional file 4). A total of 487 consecutive, unduplicated *E. coli* that were phenotypically positive for ESBL were further molecularly analyzed.

### Preparation of crude bacterial lysates and PCR mixtures

DNA was released from bacterial cells by boiling [17]. Briefly, bacteria were harvested from 1.5 mL of Luria–Bertani broth cultures by centrifugation and then resuspended in a total volume of 200 µL of molecular grade water and heated at 100 °C for 10 min. After the 10-min centrifugation, the supernatant containing bacterial DNA was collected and used for all subsequent PCR reactions. All PCR amplifications, except for the assignment of phylogenetic groups according to the revised Clermont protocol [18] were performed in a total volume of 25 μL containing 2 µL of the bacterial lysate, 12.5 μL of the PCR Master mix (Thermo Fisher Scientific, Waltham, Massachusetts, USA), and a 10 μM concentration of each primer.

### Molecular ESBL and PMQR typing

All 487 isolates were tested for the presence of *bla*_CTX-M_ group genes, *bla*_TEM_, *bla*_SHV_ [19, 20] and plasmid-mediated quinolone resistance (PMQR) genes (*qnrA*, *qnrB*, *qnrS, qnrC, qnrD, aac(6’)-Ib*, *aac(6’)-Ib-cr, oqxA*, *oqxB*, *qepA*) [21–26] using specific primers and cycling conditions described in Additional file 2. To detect the wild-type and mutated aminoglycoside acetyltransferase variants, we performed PCR amplification (primers and cycling conditions are described in Additional file 2) followed by restriction with enzyme *Taa*I (10 U/μL). Restrictions were performed in a total volume of 10 μL containing 2 μL of the PCR product, 1 μL of Tango buffer (Thermo Fisher Scientific, Waltham, Massachusetts, USA), and 0.2 μL of *Taa*I enzyme (Thermo Fisher Scientific, Waltham, Massachusetts, USA) at 65 °C for 1 h. After agarose gel electrophoresis, three fragments were observed after restriction of the wild-type allele *aac(6’)-Ib* (75, 108, and 331 bp) and four after restriction of the mutated allele *aac(6’)-Ib-cr* (75, 108, 114, and 217 bp) [27].

### Phylogenetic group assignment

For all isolates, phylogenetic groups A, B1, B2, and D were determined by multiplex PCR [28]. In addition, a multiplex PCR reaction was performed to identify eight phylogenetic groups using the revised, so-called extended quadruplex method [18]. Both methods were used to facilitate comparison with other studies. Primers and cycling conditions for both reactions are described in Additional file 2.

### Sequence types and PCR O25b typing

*E. coli* sequence types (STs) 69, 73, 95, and 131 were detected using a multiplex PCR reaction described by Doumith et al. [29] (Additional file 2). Several isolates were subjected to full 7-locus multilocus sequence typing (MLST) analysis to confirm the accuracy of the PCR method. MLST was performed according to Wirth et al. [30] using PCR primers and protocols previously reported on the *E. coli* MLST website (http://mlst.warwick.ac.uk/mlst/dbs/Ecoli) to amplify the housekeeping genes *adk*, *fumC*, *gyrB*, *icd*, *mdh*, *purA,* and *recA* (Additional file 2). The purified PCR products were sent to Microsynth AG (Balgah, Switzerland) for DNA sequencing. Sequences were analyzed for allele profiles and sequence types according to the Achtman 7 MLST scheme on the EnteroBase website.

For detection of the O-type 25b specific *pabB* gene, we followed the protocol of Clermont et al. [31] with modified cycling conditions, listed in Additional file 2 [31].

### Virulence genotyping

All 487 isolates were screened for the presence of 16 ExPEC-associated virulence factor genes encoding adhesins (*afa/dra, fimH, iha*), autotransporters (*fluA, sat, vat*), iron acquisition systems (*fyuA, iutA*), protectins (*iss, kpsMT*II*, ompT*_APEC_*, traT*), and toxins (*ehxA*, *hlyA, hlyF, usp*) by PCR using the primers and amplification procedures described in Additional file 2 [32–41].

### Clonal diversity

For genetic differentiation of isolates (which we defined as clonal groups in our study), Enterobacterial Repetitive Intergenic Consensus Polymerase Chain Reaction (ERIC-PCR) was used [42]. Primers and cycling conditions are described in Additional file 2. After agarose gel electrophoresis, banding patterns were analyzed using the BioNumerics software (version 7.6; Applied Maths NV, Sint-Martens-Latem, Belgium).

Isolates for which banding patterns could not be retrieved after several attempts of ERIC-PCR or which had weak banding patterns were not used for further analysis (n = 102). For the remaining 385 isolates, cluster analysis was performed to analyze fingerprint types, and dendrograms were generated using the unweighted pair-group method with averaging (UPGMA) based on the number of different bands with a band tolerance of 1% and optimization of 1%. The cluster analysis was followed by an advanced cluster analysis using UPGMA based on the similarity matrix without resampling.

### Statistical analysis

Statistical analysis was performed using IBM SPSS Statistics (version 25, IBM Analytics, NY). Dichotomous variables were compared using a Pearson Chi-square test and described as frequencies and percentages. All tests were two-sided, and P < 0.05 were considered statistically significant.

## Results

### ESBL-EC isolates

The majority (n = 400; 82.1%) of 487 ESBL-EC isolated from LRT samples between 2002 and 2019 were from twenty-nine different units of a large university hospital. For confidentiality, the departments of a large university hospital were assigned codes consisting of the capital letter A followed by the number. Only 15.4% (n = 75) of the isolates were from a national center of oncology (B), a general hospital (C), and some specialized outpatient and community healthcare centers located in the Central Slovenia region. 2.5% (n = 12) of the isolates were of unknown origin, all isolated between 2002 and 2004. Most of the isolates were from patients in department A6 (n = 121; 24.8%), followed by department A3 (n = 59; 12.1%), department A9 (n = 41; 8.4%), general hospital (n = 33; 6.8%), and a national center of oncology (n = 29; 6.0%). For the purposes of this study, an independent location was defined if at least 5 ESBL-EC isolates were isolated there; otherwise, isolates were assigned to a group labeled “Other Locations” (OTL).

Based on phenotypic resistance profiles, 410 (84.2%) were defined as multidrug-resistant (MDR) and 13 (2.7%) as extensively drug-resistant (XDR) strains. Testing for carbapenem resistance (imipenem, ertapenem, meropenem) revealed that only one isolate (0.2%) was resistant to ertapenem.

### Prevalence of beta-lactamase and plasmid-mediated quinolone resistance genes in ESBL-EC from LRT isolated between 2002 and 2019

*bla*_CTX-M_ genes were detected in 457 (93.8%) out of 487 phenotypically positive ESBL isolates by group-specific PCR. Detailed PCR analysis revealed that 265 (54.4%), 10 (2.1%), 377 (77.4%), and 80 (16.4%) carried *bla*_TEM_, *bla*_SHV_, group 1 and group 9 *bla*_CTX-M_ genes, respectively. 201 (41.3%) of all isolates carried *bla*_TEM_ and group 1 *bla*_CTX-M_ genes, 48 isolates (9.9%) carried *bla*_TEM_ and group 9 *bla*_CTX-M_ genes, 4 isolates (0.8%) carried *bla*_TEM_ and *bla*_SHV_ genes, and 2 isolates (0.4%) carried *bla*_SHV_ and group 1 *bla*_CTX-M_ genes. None of the 487 isolates had a combination of *bla*_SHV_ and group 9 *bla*_CTX-M_ genes. Only one (0.2%) harbored three β-lactamase genes tested, namely *bla*_TEM_, *bla*_SHV_, and group 9 *bla*_CTX-M_.

**Fig. 1:**
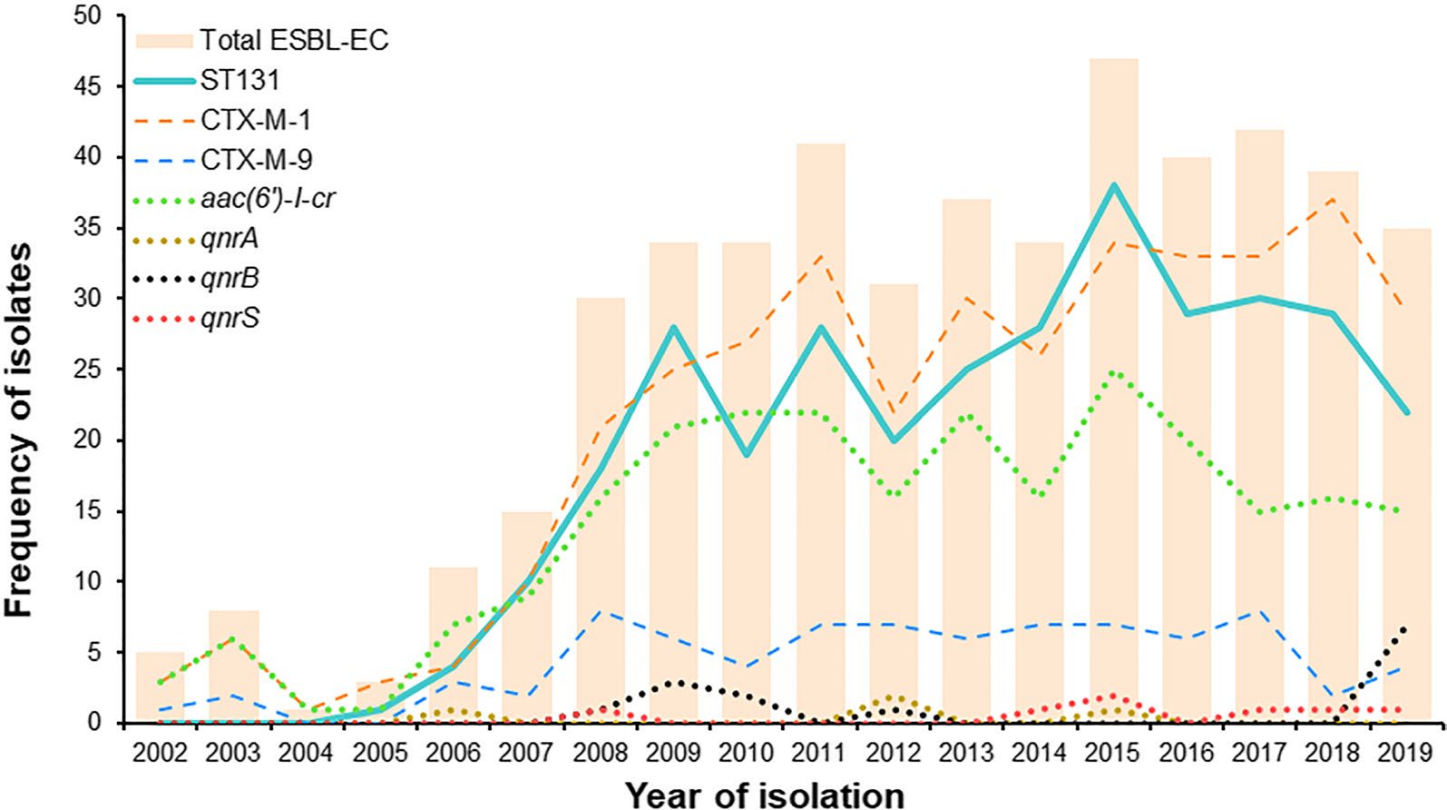
Distribution of antibiotic resistance genes and ST131 clonal group among ESBL-EC isolates over an 18-year period. Dashed lines represent the prevalence of *bla*_CTX-M_ genes from groups 1 and 9, labelled as CTX-M-1 and CTX-M-9, respectively. Dotted lines represent the prevalence of PMQR genes *aac(6’)-Ib-cr*, *qnrA*, *qnrB*, and *qnrS*. Data for *bla*_TEM_ and *bla*_SHV_ are not shown

While the percentage of isolates with *bla*_SHV_ gene was low throughout the period, the percentage of isolates with *bla*_TEM_ gene ranged from 80% (n = 4) in 2002 to 48.6% (n = 17) in 2019 (Fig. 1). The percentage of isolates, positive for group 1 *bla*_CTX-M_, ranged from 60% (n = 3) in 2002 to 82.9% (n = 29) in 2019, with a sharp increase after 2006, while the proportion of isolates, positive for group 9 *bla*_CTX-M_ genes ranged from 20% (n = 1) in 2002 to 11.4% (n = 4) in 2019. Resistance data for each isolate are presented in Additional file 1, Additional file 2, Additional file 3, and Additional file 4.

Eighteen (3.7%) of 487 isolates examined were positive for *qnr* genes. Gene *qnrA* was detected in four (0.82%) isolates, whereas each of *qnrB* and *qnrS* were detected in seven isolates (1.4%). The mutant variant of the aminoglycoside acetyltransferase allele (*aac(6’)-Ib-cr*) was detected in 253 (52%) of all isolates, whereas the wild-type allele (*aac(6’)-Ib*) was detected in only 8 (1.6%). Between 2016 and 2017, a rapid decrease in the prevalence of isolates with the mutant allele *aac(6’)-Ib-cr* from 50% (n = 20) to 35.7% (n = 15) was observed, which was not aligned with a lower percentage of CTX-M-positive ST131 isolates. Genes *oqxA*, *oqxB,* and *qepA* were not detected.

### Phylogenetic groups

ESBL-EC isolates from LRT were classified into phylogroups based on two Clermont’s phylo-typing protocols (Additional file 1, Additional file 2, Additional file 3). According to the first method described in 2000, the majority of isolates belonged to the virulent extraintestinal group B2 (n = 335; 68.8%) and to a lesser extent, to group D (n = 82; 16.8%), followed by the predominantly commensal group A (n = 58; 11.9%) and B1 isolates (n = 12; 2.5%). Compared with the revised protocol, the majority of isolates were also assigned to group B2 (n = 356; 73.1%), followed by group A (n = 34; 7%), D (n = 31; 6.4%), C (n = 28; 5.7%), F (n = 24; 4.9%), B1 (n = 11; 2.3%), and group E (n = 3; 0.6%). According to the revised protocol, the majority of isolates obtained between 2002 and 2006 were assigned to phylogenetic group C (n = 10; 35.7%), whereas the later isolates were mainly assigned to group B2 (n = 348; 75.8%). In the predominant phylogenetic group B2, we also detected the highest proportion of group 1 (n = 290; 76.9%) and group 9 *bla*_CTX-M_ genes (n = 54; 67.5%).

### Sequence types and O25*pabB* allele

PCR typing, confirmed by a complete MLST-analysis for selected isolates, revealed that 329 (67.6%) out of 487 isolates belonged to the clonal group ST131. The first five confirmed ST131 isolates were from 2005 (n = 1) and 2006 (n = 4). Thereafter, the number fluctuated between ten isolates (66.7%) in 2007 and twenty-two (62.9%) in 2019, with the lowest numbers of 18 (60%) and 19 (55.9%) recorded in 2008 and 2010, respectively, and the highest number of 38 (80.9%) isolates in 2015. Overall, more than 70% of all ESBL-EC from LRT isolated after 2006 belonged to the clonal group ST131. Only nine isolates (1.8%) were assigned to ST69, including one isolate in 2005, 2010, 2012, 2016, three in 2017 and two in 2019. Five isolates were assigned to group ST73, including one isolate in 2007, 2018, 2019, and two in 2011. Another five isolates were assigned to ST95 with one isolate in 2003, 2004, 2008, 2009, and 2010.

The *pabB* gene for the O25b-ST131 clone was detected in 380 (78%) *E. coli* ESBL isolates (Table 1). The prevalence of *pabB* in clonal groups ST131 and non-ST131 was 95.1% (n = 313) and 42.4% (n = 67) (p < 0.001), respectively.

**Table 1:** Distribution of virulence associated genes among ESBL-EC from LRT and by ST131 affiliation

VAGs^a^	Total ESBL	ST131	non-ST131	Pearson Chi-Square value (df 1)	p-value^b^
	N = 487 (100%)	N = 329 (100%)	N = 158 (100%)		
	n (%)	n (%)	n (%)		
Adhesins					
*afa/dra*	142 (29.2%)	129 (39.2%)	13 (8.2%)	49.6	< 0.001
*fimH*	450 (92.4%)	312 (94.8%)	138 (87.3%)	8.5	0.003
*iha*	336 (69%)	311 (94.5%)	25 (15.8%)	309.1	< 0.001
Autotransporters					
*fluA*	379 (77.8%)	291 (88.4%)	88 (55.7%)	66.4	< 0.001
*sat*	191 (39.2%)	165 (50.2%)	26 (16.5%)	50.8	< 0.001
*vat*	12 (2.5%)	0 (0%)	12 (7.6%)	25.6	< 0.001
Protectins					
*iss*	38 (7.8%)	3 (0.9%)	35 (22.2%)	66.9	< 0.001
*kpsMT*II	327 (67.1%)	261 (79.3%)	66 (41.8%)	68.3	< 0.001
*ompT*_APEC_	167 (34.3%)	104 (31.6%)	63 (39.9%)	3.2	< 0.001
*traT*	403 (82.8%)	288 (87.5%)	115 (72.8%)	16.3	< 0.001
Iron acquisition systems					
*fyuA*	423 (86.9%)	318 (96.7%)	105 (66.5%)	85.3	< 0.001
*iutA*	414 (85%)	320 (97.3%)	94 (59.5%)	119.5	< 0.001
Toxins					
*ehxA*	0 (0%)	0 (0%)	0 (0%)		
*hlyA*	32 (6.6%)	23 (7%)	9 (5.7%)	0.3	0.590
*hlyF*	2 (0.4%)	0 (0%)	2 (1.3%)	3.1	0.078
*usp *	347 (71.3%)	314 (95.4%)	33 (20.9%)	289.7	< 0.001
O25b serotype	380 (78%)	313 (95.1%)	67 (42.4%)	173.1	< 0.001

^a^*afa/dra* AFA-DR family adhesins, *ehxA* enterohemolysin, *fimH* type 1-fimbrial adhesin, *fluA* adhesin antigen 43, *fyuA* yersiniabactin receptor, *hlyA* hemolysin A, *hlyF* hemolysin F, *iha* adhesin-siderophore, *iss* increased serum survival, *iutA* aerobactin receptor, *kpsMT*II group II capsule, *ompT*_APEC_ avian outer membrane protease T, *sat* secreted autotransporter toxin, *traT* transfer protein, *usp* uropathogenic specific protein, VAGs virulence associated genes, *vat* vacuolating autotransporter toxin

^b^P-values (ST131 vs. non-ST131) calculated by Chi-square test are shown. P < 0.05 were considered statistically significant

### Distribution of VAGs

Among the 16 VAGs tested, *fimH* (92.4%; n = 450), *fyuA* (86.9%; n = 423), *iutA* (85%; n = 414), *traT* (82.8%; n = 403), and *fluA* (77.8%; n = 379) were the most common (Table 1). In contrast, *vat* and *iss* were confirmed in only 2.5% (n = 12) and 7.8% (n = 38), respectively.

Of all ESBL-EC isolates from LRT, 30 (6.2%) were hemolytic on sheep blood agar plates. Further PCR analysis was used to detect genes associated with hemolysis (Table 1). Thirty-two (6.6%) isolates carried gene *hlyA* and two isolates (0.4%) were positive for *hlyF*. Hemolytic isolates were all assigned to phylogenetic group B2. None of the isolates was positive for *ehxA*.

### Prevalence and distribution of VAGs in relation to the phylogenetic groups

The majority of 487 ESBL-EC (n = 356; 73.1%) belonged to phylogenetic group B2 and the prevalence of VAGs among these isolates ranged from 49 to 96%, with the exception of *afa*/*dra* (36.5%; n = 130), *ompT*_APEC_ (33.7%; n = 120), *iss* (1.4%; n = 5), and *vat* (3.1%; n = 11). The highest frequencies of VAGs in group B2 isolates were for *fyuA* (96.3%, n = 343), *fimH* (94.9%, n = 338), *iutA* (94.7%, n = 337), and *usp* (94.7%; n = 337). Furthermore, 34 (7%) ESBL-EC isolates from LRT were assigned to phylogenetic group A. The most abundant VAGs among these isolates were *fimH* (82.4%; n = 28), *traT* (79.4%; n = 27), *fyuA* (58.8%; n = 20), and *iutA* (55.9%; n = 19). Among the thirty-one isolates (6.4%), assigned to phylogenetic group D, 93.5% (n = 29) carried *fimH*, 77.4% (n = 24) carried *fyuA*, 74.2% (n = 23) carried *fluA*, and 67.7% (n = 21) carried *traT*. None of the isolates in groups A and D were positive for *vat* and none for *usp* in group D.

### Prevalence and distribution of virulence factor genes in relation to ST131

As shown in Table 1, the most common VAGs in non-ST131 isolates were *fimH* (87.3%; n = 138), *traT* (72.8%; n = 115), *fyuA* (66.5%; n = 105), *iutA* (59.5%; n = 94), and *fluA* (55.7%; n = 88). The percentage of all VAGs tested, except for *ompT*_APEC_*, vat*, and *iss*, was significantly higher in ST131 isolates compared with non-ST131 isolates, ranging up to 39.2% for *afa/dra* and 79.3% to 97.3% for *fimH*, *iha*, *fluA*, *kpsMT*II, *traT*, *fyuA, iutA*, and *usp.*

Among all ESBL-EC from LRT, the average VAGs score for the 16 virulence factor genes tested was 7.52, 18.1% (n = 88) were positive for more than 10 VAGs, and only 21.6% (n = 105) had 5 or fewer VAGs. The average number of VAGs among ST131-positive isolates was 8.63, and 5.22 in group of non-ST131 isolates.

### Clonal diversity in relation to patient location and sequence type

**Fig. 2:**
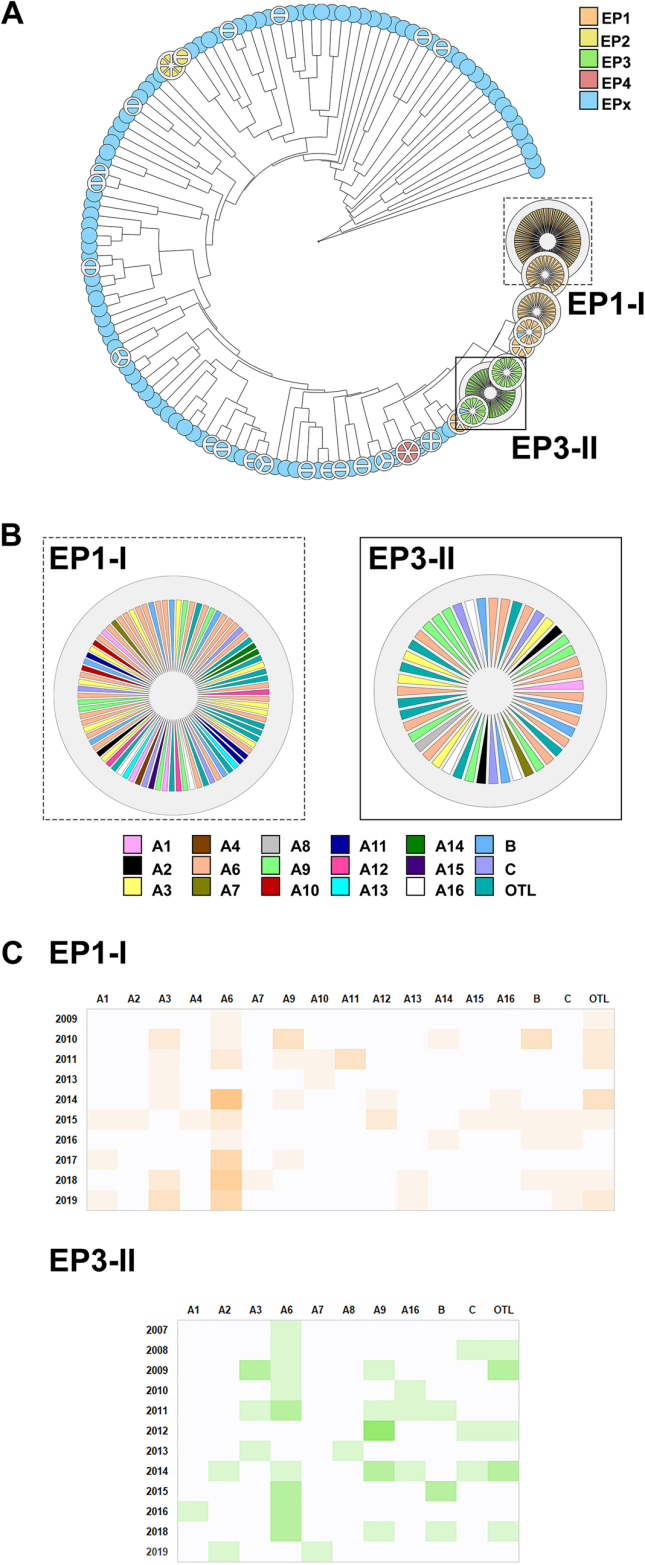
Clonal diversity of ESBL-EC isolates from LRT (n = 385). Isolates were classified into five ERIC-profile (EP) groups designated EP1, EP2, EP3, EP4, and EPx (other ERIC-profiles). **A** Circular dendrogram showing groups of isolates with similar ERIC-profiles. Two major clusters of isolates within groups EP1 and EP3, designated as EP1-I and EP3-II, are marked with a dashed and solid line, respectively. **B** Location distribution of isolates from cluster EP1-I and EP3-II. **C** Heatmap diagram showing the prevalence of isolates from cluster EP1-I and EP3-II according to location over time. Legend for **B** and **C**: Different departments of hospital A are designated as A1–A16; the national center of oncology with letter B; a general hospital with C and isolates assigned to group “Other Locations” with OTL

Based on DNA-fingerprinting patterns retrieved with ERIC-PCR and analyzed with the BioNumerics program, 385 isolates were classified into five groups designated EP1, EP2, EP3, EP4, and EPx (other ERIC-profiles) (Fig. 2A). The EPx group included isolates with unique profiles or clusters of isolates with a number of less than five. Isolates within an EP-group with identical EP profiles were gathered into clusters. Main clusters from EP1- and EP3-group were designated as I (EP1-I) and II (EP3-II), respectively (Fig. 2A).

Of the 385 isolates, 161 (41.8%) were assigned to group EP1, 10 (2.6%) to group EP2, 72 (18.7%) to group EP3, and 6 (1.6%) to group EP4. Only isolates with the EP1-group banding pattern were retrieved from all locations. Isolates with EP2-banding profile were detected only in the non-ST131 group, whereas EP3- and EP4-groups were detected only in the ST131 group, except in one isolate. The majority of EP-group 1 (96.3%; n = 155; p < 0.001), and EP-group 3 (98.6%; n = 71; p < 0.001) isolates were ST131-positive. 13.1% (n = 35) of ST131 and 85.6% (n = 101) of non-ST131 isolates were assigned to the EPx-group.

ESBL-EC from LRT with identical EP-banding profiles were isolated from different patients and different hospital departments and institutions, including remote locations. Locations of isolates from clusters EP1-I and EP3-II are shown in Fig. 2B. In addition, the prevalence of isolates from clusters EP1-I and EP3-II according to location over the sampling period is shown in Fig. 2C.

## Discussion

Our 18-year retrospective study of location-defined isolates provides important insights into the prevalence and dynamics of β-lactamase and plasmid-mediated quinolone resistance genes, the occurrence of the ST131 sequence type, and the virulence potential of ESBL-EC strains, isolated from LRT samples and identified as ESBL-EC. To the best of our knowledge, this is the first comprehensive study of the genetic diversity of *E. coli* isolates from LRT over an extended period of time.

The global spread of antibiotic resistance genes through ESBL-EC, particularly clone ST131, is a major public health concern [43, 44]. Despite the widespread distribution of ESBL-EC and its association with many infections, comparison of our data with other studies is difficult because they typically include strains from shorter sampling periods and/or fewer isolates analyzed. Long-term studies mainly include ESBL-EC isolates from bacteremia, UTIs, or various other clinical samples [9, 44–50]. In our study, the first confirmed ESBL-EC from LRT (n = 5) date back to 2002 (Fig. 1). The prevalence of ESBL-EC among all *E. coli* isolates from LRT in the large university hospital A was low in 2005 (1.4%; n = 3) but has steadily increased since then, peaking at 10.8% (n = 30) in 2019. The increasing number correlates with the global spread of the clonal group ST131, which was first identified in LRT samples in the Central Slovenia region in 2005 and accounted for 67.6% (n = 329) of all 487 analyzed ESBL-EC from LRT. After 2016, we observed a slight decrease of ESBL-EC and ST131. Data collected from the laboratory information system at the Institute of Microbiology and Immunology (IMI) also show that the percentage of ESBL-EC among LRT isolates (8.3%) in the large university hospital A was slightly higher than the percentage of all clinical ESBL-EC isolates (LRT isolates excluded; 6.2%) during the sampling period from 2005 to 2019 (Additional file 1.1). In contrast, Tang et al. (2018) described that ESBL-EC represented 57.5% of all *E. coli* isolates from sputum collected at respiratory departments in China [51].

ExPEC strains are most commonly involved in UTIs or bacteremia, but are also associated with pneumonia, which is a common nosocomial infection in patients receiving mechanical ventilation. La Combe et al. [5] described ESBL-EC, isolated between 2012 and 2014, that accounted for 8.5% of pneumonia-specific isolates, which is highly comparable to the results of our study during the same period (i.e., 8.8%). It is worth noting that the predominant sequence type complex among pneumonia-specific *E. coli* isolates in the study by La Combe et al. [5] was ST73 [5], which was detected in only 1% of isolates in the present study. However, the percentage of ESBL-EC and clonal group ST131 varies between studies due to different sampling periods and sample types [9, 45–48]. For example, in a Danish study between 2008 and 2009, only 2% ESBL producers were detected among all *E. coli* isolates (mainly urine samples), of which 44% belonged to the clonal group ST131 [48]. Furthermore, an 11-year study of bacteremic ESBL-EC isolates from Canada also showed a lower proportion of ESBL-producing isolates between 2000 and 2006 (1.7%), followed by a significant increase of ESBL-EC from 2007 (4.1%) to 2010 (13.7%). When compared to our data for the period from 2005 to 2010 in hospital A, there was a similar overall proportion of ESBL isolates (i.e., 5.8% vs. 6.4%) and also a similar percentage of ST131 among ESBL isolates (i.e., 64.2% vs. 59.4%) [49].

Gram-negative bacteria, including *E. coli*, can cause respiratory infections, associated with poor clinical outcomes due to limited treatment options [52]. Our collection of 487 isolates ESBL-EC was phenotypically tested for susceptibility to antimicrobials from eleven antimicrobial categories [15, 16]. 84.2% and 2.7% of isolates were multidrug resistant and extensively drug-resistant, respectively. Studies analyzing the prevalence of MDR and XDR among ESBL-EC respiratory isolates over a long period of time are scarce. Other studies are generally conducted on all *E. coli* isolates and examine the prevalence of ESBL and MDR among all *E. coli* isolates. Among other ExPEC strains, a high prevalence of MDR among ESBL-EC (62%) isolates, retrieved from community acquired UTIs was reported by Hassuna et al. [53]. As a result, treatment options for ESBL-EC infections are becoming restricted to last resort antimicrobials, e.g., carbapenems. However, the rate of resistance to carbapenems is steadily increasing [54].

According to the data from the literature, the first plasmid-encoded ESBLs were described in the 1980s, when strains with dominant TEM- and SHV-type enzymes were mainly associated with hospital-acquired infections. In the 2000s, ESBL-producing strains encoding a new group of ESBLs, namely CTX-M, were also recovered from community. The most widely spread CTX enzymes are CTX-M-15 and CTX-M-14 from the CTX-M-1 and M-9 groups, respectively. In the majority of European countries, the CTX-M-1 group is the most prevalent, whereas in Asian countries, the dominating group is CTX-M-9 [44, 55–59]. Consistent with this are the results of our study, as genes for group 1 CTX-M enzymes were detected in 77.4% of isolates and for group 9 in only 16.4% of all 487 isolates (Fig. 1). Among the studied respiratory ESBL-EC isolates examined, group CTX-M-1 enzymes dominated since 2006 with several peaks thereafter, while the proportion of group CTX-M-9-positive isolates was consistently low and evenly distributed over the years. Moreover, CTX-M-9-group-positive isolates were not significantly related to ST131 affiliation. While the percentage of *bla*_TEM_-positive isolates was 67.9% between 2002 and 2006 and decreased to 53.6% between 2007 and 2019, the percentage of *bla*_SHV_ and *qnr* genes was low throughout the study period. In contrast to our results, the study by La Combe et al. showed a lower percentage of group 1 *bla*_CTX-M_ genes (59.1%) and a higher percentage of group 9 *bla*_CTX-M_ genes (27.3%) in pneumonia-specific *E. coli* [5]. A 12-year study in Spain that included bacteremic ESBL-EC showed a predominance of group 1 CTX-M enzymes (54.2%; mostly CTX-M-1 and CTX-M-15 and less frequently CTX-M-32). In addition, a high percentage of group CTX-M-9 was detected (41.7%) [46].

For the treatment of bacterial infections, the most extensively used classes of antibiotics are β-lactams and quinolones. Resistance to quinolones is principally mediated by mutations in the chromosomal quinolone resistance determining region (QRDR), but may also be plasmid-mediated by *qnr*, *aac(6′)-Ib-cr*, and genes for various efflux pumps (PMQR). Although PMQR genes alone confer clinically irrelevant levels of resistance, they may indirectly influence the selection of strains with high levels of resistance [60–63]. In this study, half of the isolates (52%) were found to harbor *aac(6')-Ib-cr*, eight (1.6%) harbored *aac(6’)-Ib*, and only 18 (3.7%) isolates were positive for one of the *qnr* genes (*qnrA*, *qnrB* or *qnrS*). Several studies showed that PMQR and ESBL genes may be encoded on the same plasmid. The presence of both resistance determinants on conjugative plasmids in a pandemic clonal strain allows global spread and co-transmission of genes conferring resistance to third-generation cephalosporins (e.g. *bla*_CTX-M-15_), variable resistance to aminoglycosides (*aac(6')-Ib-cr*), and low-level resistance to ciprofloxacin. Intermediate ciprofloxacin resistance can also be detected in strains with both *qnrS* and *aac(6')-Ib-cr* genes [60–63]. In the current study, a combination of *aac(6')-Ib-cr* and *qnrS* was detected in only four isolates, two of which also harbored group 1 *bla*_CTX-M_ genes. In addition, a combination of *aac(6')-Ib-cr* and group 1 *bla*_CTX-M_ genes was confirmed in 239 (49.7%) ESBL-EC isolates and a combination of *aac(6')-Ib-cr* and group 9 *bla*_CTX-M_ genes in three isolates (0.61%). Analysis of plasmid-mediated quinolone resistance and β-lactamase genes in relation to ST131 affiliation showed that ST131 isolates were significantly more likely to carry *aac(6')-Ib-cr* and group 1 *bla*_CTX-M_ genes compared with non-ST131 isolates. Thus, 176 (53.5%) out of 329 ST131 isolates had a combination of *aac(6')-Ib-cr* and group 1 *bla*_CTX-M_ genes, in contrast to non-ST131 isolates in which both genes were detected in significantly lower percentages (39.9%; p = 0.05). Park et al. (2012) described that 36.5% ESBL-EC strains, isolated mainly from urine samples, carried the *aac(6')-Ib-cr* allele. Of the 23 *aac(6')-Ib-cr*-positive isolates, 19 and 2 isolates produced CTX-M group 1 and CTX-M group 9 enzymes, respectively. In addition, enzymes from both groups were confirmed in two isolates [64].

In general, human ExPEC strains belong to phylogenetic group B2 and D, whereas commensal and less virulent strains belong to group A or B1 [28]. In the present study, the majority of isolates belonged to phylogenetic group B2, followed by groups A and D. ST131 isolates were significantly associated with phylogenetic group B2 (99.1%; p < 0.001), and only three isolates from group ST131 were assigned to other groups (D and F). In contrast, isolates from the non-ST131 group were evenly distributed among groups A, B2, D, C, and F. These results correlate with other studies, including *E. coli* isolates, mainly from urinary tract or blood cultures [3, 9, 45, 46, 48, 49].

ExPEC strains possess a wide range of virulence-associated factors, including adhesins, toxins, iron acquisition factors, and others, that enable them to colonize and invade the host and evade the immune system, ultimately leading to extraintestinal diseases [3, 65]. Considering that the type and number of VAGs detected in our study are similar to those of other ESBL-producing ExPEC strains, we suggest that ESBL-EC isolates from LRT do not have a unique VAG profile (Table 1) [3, 9, 45, 46, 48, 49]. In our study, ST131-positive isolates have similar core virulence genes with a high prevalence of *iutA, fyuA, usp*, *fimH*, *iha*, *fluA, traT, kpsMT*II and low prevalence of *sat* and *afa/dra*. The comparison of the ST131 with the non-ST131 group isolates shows statistically significant differences, thus supporting the idea of a greater virulence potential of ST131 and confirms the results of previous studies [3, 9, 45, 46, 48, 49].

Fingerprinting by ERIC-PCR was employed to determine whether similar strains were isolated from different patients at different locations and sampling periods. Isolates from group EP1 (n = 161; 41.8%) were detected at every single location, even when the distance between some locations exceeded 50 km. The second most common profile among studied ESBL-EC from LRT was EP3 (n = 72; 18.7%), which was detected at the majority of sample locations. ERIC-profiles from the clusters EP1-I and EP3-II were detected at different locations (Fig. 2B). Of the non-ST131 isolates, 101 (85.6%) were assigned to the EPx group, indicating greater diversity compared to the ST131 group.

According to other studies, hospitals, including nursing homes, are the main reservoir of ESBL-EC and ST131. In addition, long-term hospitalization is a known risk factor for nosocomial infections [2, 7, 44, 56]. Thus, infections with ESBL-EC can be acquired both in the hospital and in the community and correlate with several risk factors, such as repeated UTIs, previous antibiotic treatment (especially cephaloporins), hospitalization, residence in nursing homes, patient age, and others [66]. Peirano and colleagues [49] showed that ST131 clones originated primarily from health care settings (i.e., 61%) with a smaller proportion acquired in hospitals (i.e., 17%) or in the community (i.e., 22%) [49]. In the present study, ESBL-EC from LRT with the same ERIC-PCR profile (e.g. EP1-I and EP3-II) were isolated from different patients, from different years, and even from different hospital departments and institutions, including remote locations, indicating the presence of “hospital-adapted” strains in the Central Slovenia region (Fig. 2C).

Recent studies showed that the number of intestinal carriers of ESBL-EC is increasing worldwide, not only in hospitalized patients but also in healthy individuals [67]. In order to confirm the presence of “hospital adapted” strains, a nationwide screening for the presence of ESBL-EC, in particular clonal group ST131, among the general Slovenian population is thus required.

## Conclusion

In our retrospective analysis, the genetic characteristics of 487 ESBL-EC isolates from LRT in the central Slovenia region were described. The dominance of phylogroup B2 and group 1 *bla*_CTX-M_ genes is related to the highly virulent and antibiotic-resistant ST131 group, which is a major public health concern as the predominant sequence type among ESBL-EC strains. Further, our results suggest that the ESBL-EC isolates from LRT do not represent a specific pathotype, but rather resemble other ESBL ExPEC isolates, and that the isolates from our long-term study may be adapted to the hospital environment. To our knowledge, this is the first longitudinal study of ESBL-EC isolated from LRT samples collected over an extensive time period.

### Supplementary Information

**Additional file 1. **Dynamics of *E. coli* isolates, resistance genes and phylogenetic groups distribution.

**Additional file 2. **Primers and conditions for PCR amplification.

**Additional file 3. **Raw data.

**Additional file 4. **Raw data for age and gender of patient, sample category and for antimicrobial susceptibility testing.

## Data Availability

Not applicable.

## Abbreviations

CLSI: Clinical and Laboratory Standards Institute
CTX-M: Cefotaxime-hydrolyzing β-lactamase–Munich
*E. coli*: *Escherichia coli*
EP: ERIC-profile
ERIC-PCR: Enterobacterial Repetitive Intergenic Consensus Polymerase Chain Reaction
ESBL: Extended-spectrum β-lactamase
ESBL-EC: Extended-spectrum β-lactamase-producing *E. coli*
EUCAST: European Committee on Antimicrobial Susceptibility Testing
ExPEC: Extraintestinal pathogenic *E. coli*
IMI: Institute of Microbiology and Immunology, Faculty of Medicine, University of Ljubljana
LRT: Lower respiratory tract
MALDI TOF MS: Matrix-assisted laser desorption/ionization time-of-flight mass-spectrometry
MLST: Multilocus sequence typing
PMQR: Plasmid-mediated quinolone resistance
QRDR: Quinolone resistance determining region
SHV: Sulfhydryl variant of the TEM enzyme
ST: Sequence type
TEM: Temoneira class A extended-spectrum β-lactamase
UPGMA: Unweighted pair-group method with averaging
UTI: Urinary tract infection
VAGs: Virulence-associated genes

## References

[1] Poolman JT, Wacker M (2016). Extraintestinal pathogenic *Escherichia coli*, a common human pathogen: challenges for vaccine development and progress in the field. J Infect Dis.

[2] Rogers BA, Sidjabat HE, Paterson DL (2011). *Escherichia coli* O25b-ST131: a pandemic, multiresistant, community-associated strain. J Antimicrob Chemother.

[3] Sarowska J, Futoma-Koloch B, Jama-Kmiecik A, Frej-Madrzak M, Ksiazczyk M, Bugla-Ploskonska G, Choroszy-Krol I (2019). Virulence factors, prevalence and potential transmission of extraintestinal pathogenic *Escherichia coli* isolated from different sources: recent reports. Gut pathogens.

[4] Rouzé A, Jaillette E, Nseir S (2018). Relationship between microaspiration of gastric contents and ventilator-associated pneumonia. Ann Transl Med.

[5] La Combe B, Clermont O, Messika J, Eveillard M, Kouatchet A, Lasocki S, Corvec S, Lakhal K, Billard-Pomares T, Fernandes R (2019). Pneumonia-specific *Escherichia coli* with Distinct phylogenetic and virulence profiles, France, 2012–2014. Emerg Infect Dis.

[6] Okimoto N, Hayashi T, Ishiga M, Nanba F, Kishimoto M, Yagi S, Kurihara T, Asaoka N, Tamada S (2010). Clinical features of Escherichia coli pneumonia. J Infect Chemother.

[7] Kózka M, Sega A, Wojnar-Gruszka K, Tarnawska A, Gniadek A (2020). Risk factors of pneumonia associated with mechanical ventilation. Int J Environ Res Public Health.

[8] Birgy A, Levy C, Bidet P, Thollot F, Derkx V, Béchet S, Mariani-Kurkdjian P, Cohen R, Bonacorsi S (2016). ESBL-producing *Escherichia coli* ST131 versus non-ST131: evolution and risk factors of carriage among French children in the community between 2010 and 2015. J Antimicrob Chemother.

[9] Cha MK, Kang CI, Kim SH, Cho SY, Ha YE, Wi YM, Chung DR, Peck KR, Song JH (2016). Comparison of the microbiological characteristics and virulence factors of ST131 and non-ST131 clones among extended-spectrum β-lactamase-producing *Escherichia coli* causing bacteremia. Diagn Microbiol Infect Dis.

[10] Luna-Pineda VM, Ochoa SA, Cruz-Córdova A, Cázares-Domínguez V, Reyes-Grajeda JP, Flores-Oropeza MA, Arellano-Galindo J, Hernández-Castro R, Flores-Encarnación M, Ramírez-Vargas A (2018). Features of urinary *Escherichia coli* isolated from children with complicated and uncomplicated urinary tract infections in Mexico. PLoS ONE.

[11] Peirano G, Pitout JDD (2019). Extended-Spectrum β-Lactamase-Producing enterobacteriaceae: update on molecular epidemiology and treatment options. Drugs.

[12] CLSI (2019). Performance standards for antimicrobial susceptibility testing.

[13] Breakpoint tables for interpretation of MICs and zone diameters. Versions 4.0 to 9.0. https://www.eucast.org/clinical_breakpoints/ Accessed 29 Nov 2022

[14] Guidelines for detection of resistance mechanisms and specific resistances of clinical and/or epidemiological importance. Version 2.0. pp. 1–43. http://www.eucast.org/resistance_mechanisms/ Accessed 29 Nov 2022

[15] Magiorakos AP, Srinivasan A, Carey RB, Carmeli Y, Falagas ME, Giske CG, Harbarth S, Hindler JF, Kahlmeter G, Olsson-Liljequist B (2012). Multidrug-resistant, extensively drug-resistant and pandrug-resistant bacteria: an international expert proposal for interim standard definitions for acquired resistance. Clin Microbiol Infect.

[16] Wolfensberger A, Kuster SP, Marchesi M, Zbinden R, Hombach M (2019). The effect of varying multidrug-resistence (MDR) definitions on rates of MDR gram-negative rods. Antimicrob Resist Infect Control.

[17] Le Bouguenec C, Archambaud M, Labigne A (1992). Rapid and specific detection of the *pap*, *afa*, and *sfa* adhesin-encoding operons in uropathogenic *Escherichia coli* strains by polymerase chain reaction. J Clin Microbiol.

[18] Clermont O, Christenson JK, Denamur E, Gordon DM (2013). The Clermont *Escherichia coli* phylo-typing method revisited: improvement of specificity and detection of new phylo-groups. Environ Microbiol Rep.

[19] Dallenne C, Da Costa A, Decré D, Favier C, Arlet G (2010). Development of a set of multiplex PCR assays for the detection of genes encoding important beta-lactamases in Enterobacteriaceae. J Antimicrob Chemother.

[20] Woodford N, Fagan EJ, Ellington MJ (2006). Multiplex PCR for rapid detection of genes encoding CTX-M extended-spectrum (beta)-lactamases. J Antimicrob Chemother.

[21] Cattoir V, Poirel L, Nordmann P (2008). Plasmid-mediated quinolone resistance pump QepA2 in an *Escherichia coli* isolate from France. Antimicrob Agents Chemother.

[22] Cattoir V, Poirel L, Rotimi V, Soussy CJ, Nordmann P (2007). Multiplex PCR for detection of plasmid-mediated quinolone resistance *qnr* genes in ESBL-producing enterobacterial isolates. J Antimicrob Chemother.

[23] Cattoir V, Weill FX, Poirel L, Fabre L, Soussy CJ, Nordmann P (2007). Prevalence of *qnr* genes in *Salmonella* in France. J Antimicrob Chemother.

[24] Hansen LH, Sørensen SJ, Jørgensen HS, Jensen LB (2005). The prevalence of the OqxAB multidrug efflux pump amongst olaquindox-resistant *Escherichia coli* in pigs. Microb Drug Resist.

[25] Kraychete GB, Botelho LA, Campana EH, Picão RC, Bonelli RR (2016). Updated multiplex PCR for detection of all six plasmid-mediated *qnr* Gene families. Antimicrob Agents Chemother.

[26] Ni Q, Tian Y, Zhang L, Jiang C, Dong D, Li Z, Mao E, Peng Y (2016). Prevalence and quinolone resistance of fecal carriage of extended-spectrum β-lactamase-producing *Escherichia coli* in 6 communities and 2 physical examination center populations in Shanghai. China Diagn Microbiol Infect Dis.

[27] Ambrozic Avgustin J, Keber R, Zerjavic K, Orazem T, Grabnar M (2007). Emergence of the quinolone resistance-mediating gene *aac(6')-Ib-cr* in extended-spectrum-beta-lactamase-producing *Klebsiella* isolates collected in Slovenia between 2000 and 2005. Antimicrob Agents Chemother.

[28] Clermont O, Bonacorsi S, Bingen E (2000). Rapid and simple determination of the *Escherichia coli* phylogenetic group. Appl Environ Microbiol.

[29] Doumith M, Day M, Ciesielczuk H, Hope R, Underwood A, Reynolds R, Wain J, Livermore DM, Woodford N (2015). Rapid identification of major *Escherichia coli* sequence types causing urinary tract and bloodstream infections. J Clin Microbiol.

[30] Wirth T, Falush D, Lan R, Colles F, Mensa P, Wieler LH, Karch H, Reeves PR, Maiden MC, Ochman H (2006). Sex and virulence in *Escherichia coli*: an evolutionary perspective. Mol Microbiol.

[31] Clermont O, Dhanji H, Upton M, Gibreel T, Fox A, Boyd D, Mulvey MR, Nordmann P, Ruppé E, Sarthou JL (2009). Rapid detection of the O25b-ST131 clone of *Escherichia coli* encompassing the CTX-M-15-producing strains. J Antimicrob Chemother.

[32] Johnson JR, Russo TA, Tarr PI, Carlino U, Bilge SS, Vary JC, Stell AL (2000). Molecular epidemiological and phylogenetic associations of two novel putative virulence genes, iha and iroN (*E. coli*), among *Escherichia coli* isolates from patients with urosepsis. Infect Immun.

[33] Johnson JR, Stapleton AE, Russo TA, Scheutz F, Brown JJ, Maslow JN (1997). Characteristics and prevalence within serogroup O4 of a J96-like clonal group of uropathogenic *Escherichia coli* O4:H5 containing the class I and class III alleles of *papG*. Infect Immun.

[34] Johnson JR, Stell AL (2000). Extended virulence genotypes of *Escherichia coli* strains from patients with urosepsis in relation to phylogeny and host compromise. J Infect Dis.

[35] Johnson TJ, Wannemuehler Y, Doetkott C, Johnson SJ, Rosenberger SC, Nolan LK (2008). Identification of minimal predictors of avian pathogenic *Escherichia coli* virulence for use as a rapid diagnostic tool. J Clin Microbiol.

[36] Parham NJ, Pollard SJ, Desvaux M, Scott-Tucker A, Liu C, Fivian A, Henderson IR (2005). Distribution of the serine protease autotransporters of the Enterobacteriaceae among extraintestinal clinical isolates of *Escherichia coli*. J Clin Microbiol.

[37] Ruiz J, Navia MM, Vila J, Gascón J (2002). Prevalence of the sat gene among clinical isolates of Shigella spp. causing travelers' diarrhea: geographical and specific differences. J Clin Microbiol.

[38] Starčič Erjavec M, Palandačić A, Žgur-Bertok D, Ambrožič AJ (2011). Genetic background of uropathogenic *Escherichia coli* isolates from Slovenia in relation to fluoroquinolone and sulfamethoxazole/trimethoprim resistance. Acta Biol Slov.

[39] Trkov M, Dovečar D, Paragi M, Avguštin AJ (2008). Phylogenetic grouping of *Escherichia coli* isolates from patients’ stool samples with diarrhoea. Clin Microbiol Infect.

[40] Vadnov M, Barbič D, Žgur-Bertok D, Erjavec MS (2017). Escherichia coli isolated from feces of brown bears (
* Ursus arctos
*
) have a lower prevalence of human extraintestinal pathogenic
* E. coli
* virulence-associated genes. Can J Vet Res.

[41] Yamamoto S, Terai A, Yuri K, Kurazono H, Takeda Y, Yoshida O (1995). Detection of urovirulence factors in *Escherichia coli* by multiplex polymerase chain reaction. FEMS Immunol Med Microbiol.

[42] Versalovic J, Koeuth T, Lupski JR (1991). Distribution of repetitive DNA sequences in eubacteria and application to fingerprinting of bacterial genomes. Nucleic Acids Res.

[43] Lindblom A, Kiszakiewicz C, Kristiansson E, Yazdanshenas S, Kamenska N, Karami N, Åhrén C (2022). The impact of the ST131 clone on recurrent ESBL-producing *E. coli* urinary tract infection: a prospective comparative study. Sci Rep.

[44] Nicolas-Chanoine MH, Bertrand X, Madec JY (2014). *Escherichia coli* ST131, an intriguing clonal group. Clin Microbiol Rev.

[45] Kang CI, Cha MK, Kim SH, Ko KS, Wi YM, Chung DR, Peck KR, Lee NY, Song JH (2013). Clinical and molecular epidemiology of community-onset bacteremia caused by extended-spectrum β-lactamase-producing *Escherichia col*i over a 6-year period. J Korean Med Sci.

[46] Mamani R, Flament-Simon SC, García V, Mora A, Alonso MP, López C, García-Meniño I, Díaz-Jiménez D, Blanco JE, Blanco M (2019). Sequence types, clonotypes, serotypes, and virotypes of extended-spectrum β-lactamase-producing *Escherichia coli* causing bacteraemia in a Spanish hospital over a 12-year period (2000 to 2011). Front Microbiol.

[47] Muller A, Gbaguidi-Haore H, Cholley P, Hocquet D, Sauget M, Bertrand X (2021). Hospital-diagnosed infections with *Escherichia coli* clonal group ST131 are mostly acquired in the community. Sci Rep.

[48] Olesen B, Hansen DS, Nilsson F, Frimodt-Møller J, Leihof RF, Struve C, Scheutz F, Johnston B, Krogfelt KA, Johnson JR (2013). Prevalence and characteristics of the epidemic multiresistant Escherichia coli ST131 clonal group among extended-spectrum beta-lactamase-producing
* E. coli
* isolates in Copenhagen, Denmark. J Clin Microbiol.

[49] Peirano G, van der Bij AK, Gregson DB, Pitout JD (2012). Molecular epidemiology over an 11-year period (2000 to 2010) of extended-spectrum β-lactamase-producing *Escherichia coli* causing bacteremia in a centralized Canadian region. J Clin Microbiol.

[50] Mahazu S, Sato W, Ayibieke A, Prah I, Hayashi T, Suzuki T, Iwanaga S, Ablordey A, Saito R (2022). Insights and genetic features of extended-spectrum beta-lactamase producing Escherichia coli isolates from two hospitals in Ghana. Sci Rep.

[51] Tang X, Xiao M, Zhuo C, Xu Y, Zhong N (2018). Multi-level analysis of bacteria isolated from inpatients in respiratory departments in China. J Thorac Dis.

[52] Reynolds D, Burnham JP, Vazquez Guillamet C, McCabe M, Yuenger V, Betthauser K, Micek ST, Kollef MH (2022). The threat of multidrug-resistant/extensively drug-resistant Gram-negative respiratory infections: another pandemic. Eur Respir Rev.

[53] Hassuna NA, Khairalla AS, Farahat EM, Hammad AM, Abdel-Fattah M (2020). Molecular characterization of Extended-spectrum β lactamase- producing E. coli recovered from community-acquired urinary tract infections in Upper Egypt. Sci Rep.

[54] Gong L, Tang N, Chen D, Sun K, Lan R, Zhang W, Zhou H, Yuan M, Chen X, Zhao X (2020). A nosocomial respiratory infection outbreak of carbapenem-resistant Escheric hia coli ST131 with multiple transmissible bla (KPC-2) carrying plasmids. Front Microbiol.

[55] Bevan ER, Jones AM, Hawkey PM (2017). Global epidemiology of CTX-M β-lactamases: temporal and geographical shifts in genotype. J Antimicrob Chemother.

[56] Burke L, Humphreys H, Fitzgerald-Hughes D (2012). The revolving door between hospital and community: extended-spectrum beta-lactamase-producing *Escherichia coli* in Dublin. J Hosp Infect.

[57] Livermore DM, Canton R, Gniadkowski M, Nordmann P, Rossolini GM, Arlet G, Ayala J, Coque TM, Kern-Zdanowicz I, Luzzaro F (2007). CTX-M: changing the face of ESBLs in Europe. J Antimicrob Chemother.

[58] Pfeifer Y, Cullik A, Witte W (2010). Resistance to cephalosporins and carbapenems in Gram-negative bacterial pathogens. Int J Med Microbiol IJMM.

[59] Castanheira M, Simner PJ, Bradford PA (2021). Extended-spectrum β-lactamases: an update on their characteristics, epidemiology and detection. JAC Antimicrob Resist.

[60] Bodendoerfer E, Marchesi M, Imkamp F, Courvalin P, Böttger EC, Mancini S (2020). Co-occurrence of aminoglycoside and β-lactam resistance mechanisms in aminoglycoside- non-susceptible *Escherichia coli* isolated in the Zurich area, Switzerland. Int J Antimicrob Agents.

[61] Harajly M, Khairallah MT, Corkill JE, Araj GF, Matar GM (2010). Frequency of conjugative transfer of plasmid-encoded ISEcp1 - *bla*_CTX-M-15_ and *aac(6')-lb-cr* genes in Enterobacteriaceae at a tertiary care center in Lebanon - role of transferases. Ann Clin Microbiol Antimicrob.

[62] Machuca J, Ortiz M, Recacha E, Díaz-De-Alba P, Docobo-Perez F, Rodríguez-Martínez JM, Pascual Á (2016). Impact of *AAC(6')-Ib-c*r in combination with chromosomal-mediated mechanisms on clinical quinolone resistance in *Escherichia coli*. J Antimicrob Chemother.

[63] Salah FD, Soubeiga ST, Ouattara AK, Sadji AY, Metuor-Dabire A, Obiri-Yeboah D, Banla-Kere A, Karou S, Simpore J (2019). Distribution of quinolone resistance gene (qnr) in ESBL-producing *Escherichia coli* and *Klebsiella* spp. in Lomé, Togo. Antimicrob Resist Infect Control.

[64] Park KS, Kim MH, Park TS, Nam YS, Lee HJ, Suh JT (2012). Prevalence of the plasmid-mediated quinolone resistance genes, *aac(6')-Ib-cr*, *qepA*, and *oqxAB* in clinical isolates of extended-spectrum β-lactamase (ESBL)-producing *Escherichia coli* and *Klebsiella pneumoniae* in Korea. Ann Clin Lab Sci.

[65] Kim B, Kim JH, Lee Y (2022). Virulence factors associated with escherichia coli bacteremia and urinary tract infection. Ann Lab Med.

[66] Pitout JD, Laupland KB (2008). Extended-spectrum beta-lactamase-producing Enterobacteriaceae: an emerging public-health concern. Lancet Infect Dis.

[67] Bezabih YM, Sabiiti W, Alamneh E, Bezabih A, Peterson GM, Bezabhe WM, Roujeinikova A (2021). The global prevalence and trend of human intestinal carriage of ESBL-producing *Escherichia coli* in the community. J Antimicrob Chemother.

